# Efficacy and Safety of Armolipid Plus^®^: An Updated PRISMA Compliant Systematic Review and Meta-Analysis of Randomized Controlled Clinical Trials

**DOI:** 10.3390/nu13020638

**Published:** 2021-02-16

**Authors:** Arrigo F. G. Cicero, Cormac Kennedy, Tamara Knežević, Marilisa Bove, Coralie M. G. Georges, Agnė Šatrauskienė, Peter P. Toth, Federica Fogacci

**Affiliations:** 1 Hypertension and Atherosclerosis Research Group, Medical and Surgical Sciences Department, Sant’Orsola-Malpighi University Hospital, 40138 Bologna, Italy; marilisa.bove@aosp.bo.it (M.B.); federica.fogacci@studio.unibo.it (F.F.); 2 IRCCS Azienda Ospedaliero-Universitaria di Bologna, 40138 Bologna, Italy; 3 Italian Nutraceutical Society (SINut), 40138 Bologna, Italy; 4 Department of Pharmacology and Therapeutics, Trinity College Dublin and St James Hospital, Dublin 8, Ireland; kennec30@tcd.ie; 5 Department of Nephrology, Hypertension, Dialysis and Transplantation, University Hospital Centre Zagreb, 10000 Zagreb, Croatia; tknezev2@kbc-zagreb.hr; 6 Department of Cardiology, Cliniques Universitaires Saint-Luc, Université Catholique de Louvain, 1200 Brussels, Belgium; coralie.georges@uclouvain.be; 7 Faculty of Medicine, Vilnius University, LT-03101 Vilnius, Lithuania; agne.satrauskiene@santa.it; 8 Vilnius University Hospital Santariškiu Klinikos, LT-08661 Vilnius, Lithuania; 9 CGH Medical Center, Sterling, IL 61081, USA; peter.toth@cghmc.com; 10 Cicarrone Center for the Prevention of Cardiovascular Disease, Johns Hopkins University School of Medicine, Baltimore, MD 21205, USA

**Keywords:** Armolipid Plus^®^, red yeast rice, berberine, nutraceutical, supplementation, lipids, blood pressure, fasting plasma glucose

## Abstract

Armolipid Plus^®^ is a multi-constituent nutraceutical that claims to improve lipid profiles. The aim of this PRISMA compliant systematic review and meta-analysis was to globally evaluate the efficacy and safety of Armolipid Plus^®^ on the basis of the available randomized, blinded, controlled clinical trials (RCTs). A systematic literature search in several databases was conducted in order to identify RCTs assessing the efficacy and safety of dietary supplementation with Armolipid Plus^®^. Two review authors independently identified 12 eligible studies (1050 included subjects overall) and extracted data on study characteristics, methods, and outcomes. Meta-analysis of the data suggested that dietary supplementation with Armolipid Plus^®^ exerted a significant effect on body mass index (mean difference (MD) = −0.25 kg/m^2^, *p* = 0.008) and serum levels of total cholesterol (MD = −25.07 mg/dL, *p* < 0.001), triglycerides (MD = −11.47 mg/dL, *p* < 0.001), high-density lipoprotein cholesterol (MD = 1.84 mg/dL, *p* < 0.001), low-density lipoprotein cholesterol (MD = −26.67 mg/dL, *p* < 0.001), high sensitivity C reactive protein (hs-CRP, MD = −0.61 mg/L, *p* = 0.022), and fasting glucose (MD = −3.52 mg/dL, *p* < 0.001). Armolipid Plus^®^ was well tolerated. This meta-analysis demonstrates that dietary supplementation with Armolipid Plus^®^ is associated with clinically meaningful improvements in serum lipids, glucose, and hs-CRP. These changes are consistent with improved cardiometabolic health.

## 1. Introduction

Atherosclerosis cardiovascular diseases (ASCVD) are the leading cause of mortality worldwide, and the main cause of death in persons under 75 years old in Western countries, with a huge social and economic impact [1]. Pooling data from 204 countries, the Global Burden of Disease (GBD) Study recently showed that prevalent cases of total CVD nearly doubled from 271 million in 1990 to 523 million in 2019, and the number of CVD deaths steadily increased from 12.1 million in 1990, reaching 18.6 million in 2019 [2].

High serum levels of low-density lipoprotein cholesterol (LDL-C) are the most important risk factor for the development of ASCVD [3]. The American Heart Association (AHA) 2016 update on heart disease and stroke statistics verified that only 75.7% of US children and 46.6% of US adults have total cholesterol (TC) within the advised ranges (< 170 mg/dL for untreated children and < 200 mg/dL for untreated adults), with comparable rates for other Western countries [4,5].

To reach the LDL-C target, the international guidelines recommend lifestyle changes and lipid-lowering therapy depending on the severity of dyslipidemia and global CV risk [6,7]. Specific lifestyle interventions for hypercholesterolemia include a diet low in saturated fat, moderate to high-intensity physical activity, smoking cessation, as well as weight loss for overweight and obese patients [8,9]. If maintained over the long term, these lifestyle modifications can reduce LDL-C by 5% to 15% and improve ASCVD risk [10]. However, patients unable to reach their target LDL-C goals through lifestyle interventions can consider using lipid-lowering nutraceuticals [11], as also suggested by the International Lipid Expert Panel [12].

Nutraceuticals with a detectable lipid-lowering effect can be divided into natural inhibitors of hepatic cholesterol synthesis, inhibitors of intestinal cholesterol absorption, and enhancers of the excretion of LDL-C on the basis of their mechanisms of action [12]. However, the lipid-lowering effect of most nutraceuticals occurs through multiple mechanisms. The possibility that they act synergistically on multiple stages of lipid-induced vascular damage makes them potential candidates for improving the lipid-lowering effects when used in combination with diet, medications, or other nutraceuticals [13].

Armolipid Plus^®^ is a widely tested and used proprietary formulation of six naturally occurring substances containing red yeast extract (200 mg, corresponding to 3 mg of monacolin K), policosanols (10 mg), and berberine (500 mg), in addition to folic acid (0.2 mg), astaxanthin (0.5 mg), and coenzyme Q10 (2 mg), with a detectable effect on serum lipids, blood pressure (BP), fasting plasma glucose (FPG), and several markers of insulin resistance with a good safety profile [14].

Given the increasing number of good quality studies on this nutraceutical combination, the aim of our systematic review and meta-analysis was to evaluate the efficacy and safety of Armolipid Plus^®^ on the basis of the available randomized, blinded, controlled clinical trials.

## 2. Materials and Methods

The study was designed according to guidelines inthe 2009 preferred reporting items for systematic reviews and meta-analysis (PRISMA) statement [15], and was registered in the PROSPERO database (Registration number CRD42020212600). Due to the study design (meta-analysis), neither institutional review board (IRB) approval nor patient informed consent were required.

### 2.1. Search Strategy

PubMed, EMBASE, SCOPUS, Google Scholar, Web of Science by Clarivate, and ClinicaTrial.gov (accessed on 1 February 2021) databases were searched, with no language restriction, using the following search terms: “Armolipid Plus^®^” AND (“Cholesterol” OR “LDL” OR “Triglycerides” OR “Body mass index” OR “BMI” OR “Plasma Glucose” OR “Glycemia” OR “Insulin”). The wild-card term “*” was used to increase the sensitivity of the search strategy, which was limited to studies in humans. The reference list of identified papers was manually checked for additional relevant articles. In particular, additional searches for potential trials included the references of review articles on the topic of the meta-analysis and relevant abstracts from selected congresses. The literature was searched from inception to 3 February 2021.

All paper abstracts were screened by two reviewers (F.F. and A.F.G.C.) in an initial process to remove ineligible articles. The remaining articles were obtained in full text and assessed again by the same two researchers, who evaluated each article independently and carried out data extraction and quality assessment. Disagreements were resolved by discussion with a third party.

### 2.2. Study Selection Criteria

Original studies were included if they met the following criteria: (i) being a clinical trial with either a multicenter or single-center design, (ii) having an appropriate controlled design for Armolipid Plus^®^, (iii) investigating the effect of Armolipid Plus^®^ on plasma lipids, (iv) testing the safety of Armolipid Plus^®^, and (v) reporting all the adverse events that occurred during the supplementation.

Exclusion criteria included the following: (i) lack of a control group for Armolipid Plus^®^ administration, (ii) lack of blinding, (iii) lack of sufficient information about plasma lipids at baseline or follow-up, and (iv) lack of sufficient information about the prevalence and specification of adverse events. Studies were also excluded if they contained overlapping subjects with other studies.

### 2.3. Data Extraction

Data abstracted from the eligible studies were: (i) first author’s name; (ii) year of publication; (iii) study design; (iv) main inclusion criteria and underlying disease; (v) treatment duration; (vi) study groups; (vii) number of participants in the active and control group; (viii) background lipid-lowering treatment; (ix) age and sex of study participants; (x) weight, body mass index (BMI), waist circumference, systolic BP (SBP), diastolic BP (DBP), TC, triglycerides (TG), high-density lipoprotein cholesterol (HDL-C), LDL-C, aspartate aminotransferase (AST), alanine aminotransferase (ALT), creatine phosphokinase (CPK), FPG, fasting plasma insulin (FPI), homeostatic model assessment for insulin resistance (HOMA-IR) and high sensitivity C reactive protein (hs-CRP) at baseline; and (xi) discontinuation of treatment and adverse events occurred during the trials. All data extraction and database typing were reviewed by the principal investigator (A.F.G.C.) before the final analysis, and doubts were resolved by mutual agreement among the authors.

### 2.4. Quality Assessment

A systematic assessment of risk of bias in the included studies was performed using the Cochrane criteria [16]. The following items were used: adequacy of sequence generation, allocation concealment, blind addressing of dropouts (incomplete outcome data), selective outcome reporting, and other probable sources of bias [17]. Risk-of-bias assessment was performed independently by 2 reviewers (F.F. and A.F.G.C.); disagreements were resolved by a consensus-based discussion.

### 2.5. Data Synthesis

Meta-analysis was entirely conducted using Comprehensive Meta-Analysis (CMA) V3 software (Biostat, NJ) [18].

Net changes in the investigated parameters (change scores) were calculated by subtracting the value at baseline from the one after intervention, in the active-treated group and in the control one. All values were collated as mean change from baseline. Standard deviations (SDs) of the mean difference were obtained as follows, as reported by Follman et al.: SD = square root [(SD_pre-treatment_)^2^ + (SD_post-treatment_)^2^ − (2R × SD_pre-treatment_ × SD_post-treatment_)], assuming a correlation coefficient (R) = 0.5 [19]. If the outcome measures were reported in median and range (or 95% confidence interval (CI)), mean and SD values were estimated using the method described by Wan et al. [20]. The findings of the included studies were combined using a fixed-effect model or a random-effect model (using the DerSimonian–Laird method) and the generic inverse variance method based on the level of inter-study heterogeneity, which was quantitatively assessed using the Higgins index (I^2^) [21]. For continuous parameters, effect sizes were expressed as absolute mean differences (MD) and 95%CI, standardized by the change score in SD. For treatment emergent adverse events, odd ratios (OR) and 95%CI intervals were calculated using the Mantel–Haenszel method [22]. A safety analysis was performed by excluding studies with zero events in both arms. If one or more outcomes could not be extracted from a study, the study was removed only from the analysis involving those outcomes. Adverse events were considered for the analysis only if they occurred in at least two of the included clinical trials.

In order to evaluate the influence of each study on the overall effect size, a sensitivity analysis was conducted using the leave-one-out method (i.e., removing one study at a time and repeating the analysis) [23]. Two-sided *p*-values ≤ 0.05 were considered statistically significant for all tests.

### 2.6. Publication Biases

Potential publication biases were explored using a visual inspection of Begg’s funnel plot asymmetry, Begg’s rank correlation test, and Egger’s weighted regression test [24]. The Duval and Tweedie “trim and fill” method was used to adjust the analysis for the effects of publication biases [25]. Two-sided *p*-values < 0.05 were considered statistically significant.

## 3. Results

### 3.1. Flow and Characteristics of the Included Studies

**Figure 1:**
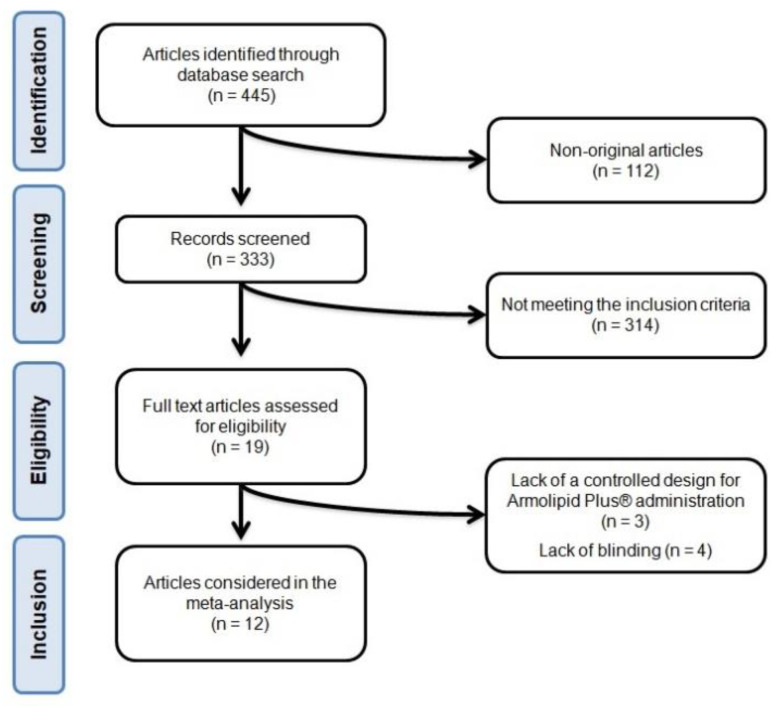
Flow chart of the number of studies identified and included in the systematic review.

After database searches were performed according to inclusion and exclusion criteria, 445 published articles were identified, and the abstracts were reviewed. Of these, 112 were excluded because they were not original articles. Another 314 were eliminated because they did not meet the inclusion criteria. Thus, 19 articles were carefully assessed and reviewed. An additional 7 studies were excluded because of a lack of a controlled design for Armolipid Plus^®^ administration (*n* = 3) or lack of blinding (*n* = 4). Finally, 12 studies were eligible and included in the meta-analysis [26,27,28,29,30,31,32,33,34,35,36,37]. The study selection process is shown in Figure 1.

Data were pooled from 12 clinical trials comprising 24 treatment arms, which included 1050 subjects, with 544 in the actively treated arm and 536 in the control one.

Eligible studies were published between 2010 and 2020. Follow-up periods ranged between 4 weeks and 12 months. All selected trials were designed with parallel groups and were multicenter [29,35,37] or single-center [26,27,28,30,31,32,33,34,36] clinical studies. Enrolled subjects were patients in primary prevention for CVD [28,29,30,32,36], patients with documented coronary artery disease (CAD) [33], with a metabolic syndrome [26,30,34,35,36], or with a good status of health [28,37]. The baseline characteristics of the evaluated studies are summarized in Table 1 and Table 2.

### 3.2. Risk of Bias Assessment

Almost all of the included studies were characterized by sufficient information regarding sequence generation, allocation concealment, and personal and outcome assessments. All showed low risk of bias because of incomplete outcome data and selective outcome reporting. Details of the quality of bias assessment are reported in Table 3.

### 3.3. Effect of Armolipid Plus^®^ on Anthropometric Measures, Blood Pressure, Serum Lipids, and Other Metabolic Parameters

**Figure 2:**
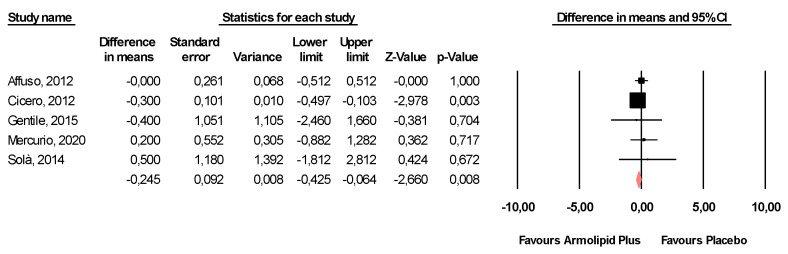
Forest plot displaying mean differences and 95% confidence intervals for the impact of the supplementation with Armolipid Plus^®^ on BMI.

**Figure 3:**
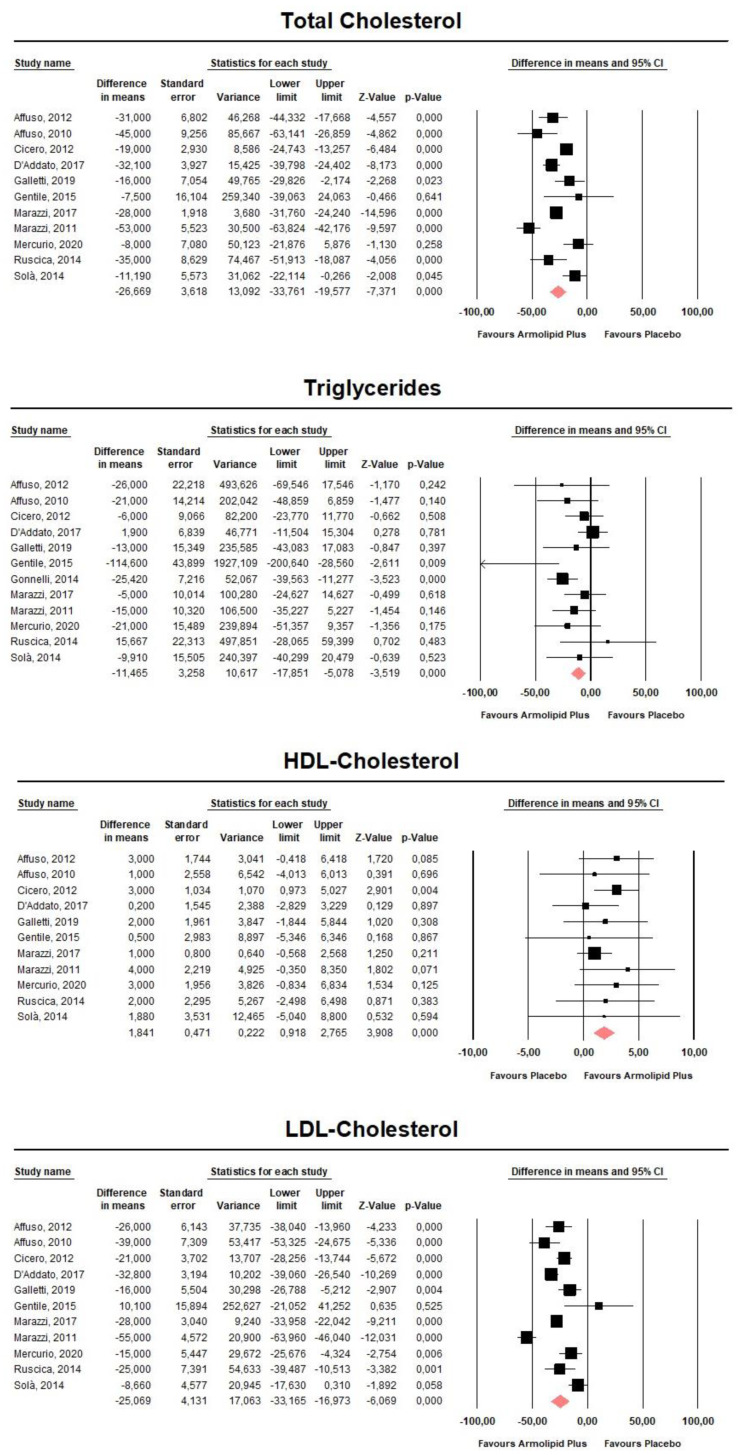
Forest plot displaying mean differences and 95% confidence intervals for the impact of the supplementation with Armolipid Plus^®^ on serum levels of TC, TG, HDL-C, and LDL-C.

**Figure 4:**
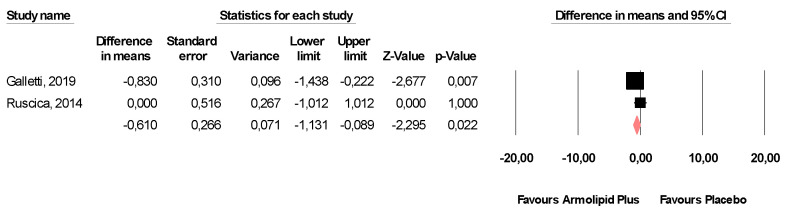
Forest plot displaying mean differences and 95% confidence intervals for the impact of the supplementation with Armolipid Plus^®^ on serum levels of hs-CRP.

**Figure 5:**
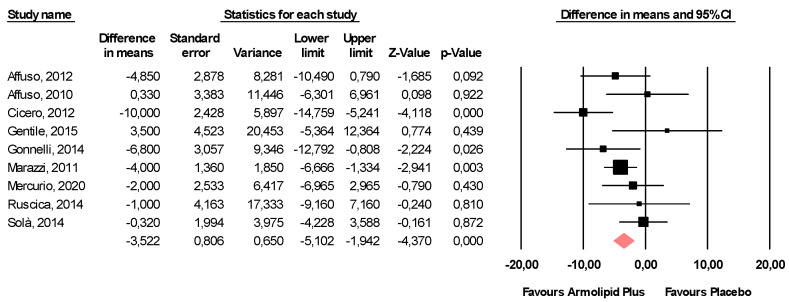
Forest plot displaying mean differences and 95% confidence intervals for the impact of the supplementation with Armolipid Plus^®^ on FPG.

Meta-analysis of the data suggested that Armolipid Plus^®^ supplementation exerted a significant effect on BMI (MD = −0.25 kg/m^2^, 95%CI(−0.43,−0.06) Kg/m^2^, *p* = 0.008; I^2^ = 0%) (Figure 2) and serum levels of TC (MD = −25.07 mg/dL, 95%CI(−33.17,−16.97) mg/dL, *p* < 0.001; I^2^ = 87%), TG (MD = −11.47 mg/dL, 95%CI(−17.85,−5.08) mg/dL, *p* < 0.001; I^2^ = 34%), HDL-C (MD = 1.84 mg/dL, 95%CI(0.92,2.77) mg/dL, *p* < 0.001; I^2^ = 0%), LDL-C (MD = −26.67 mg/dL, 95%CI(−33.76,−19.58) mg/dL, *p* < 0.001; I^2^ = 82%) (Figure 3), hs-CRP (MD = −0.61 mg/L, 95%CI(−1.13,−0.09) mg/L, *p* = 0.022; I^2^ = 47%) (Figure 4), FPG (MD = −3.52 mg/dL, 95%CI(−5.1,−1.94) mg/dL, *p* < 0.001; I^2^ = 49%) (Figure 5), without affecting weight (MD = −0.89 kg, 95%CI(−4.60,2.82) kg, *p* = 0.638; I^2^ = 0%), waist circumference (MD = −0.5 cm, 95%CI(−3.17,2.17) cm, *p* = 0.714; I^2^ = 0%) (Figure S1), SBP (MD =− 0.57 mmHg, 95%CI(−3.2,2.06) mmHg, *p* = 0.670; I^2^ = 12%), DBP (MD = −0.89 mmHg, 95%CI(−2.61,0.83) mmHg, *p* = 0.312; I^2^ = 0%) (Figure S2), FPI (MD = −0.58 mU/L, 95%CI(−1.24,0.09), *p* = 0.091; I^2^ = 30%), and HOMA-IR (MD = −0.09, 95%CI(−0.44,0.26), *p* = 0.599; I^2^ = 61%) (Figure S3).

The effect sizes were robust in the leave-one-out sensitivity analysis and not mainly driven by a single study (data not shown).

A visual inspection of Begg’s funnel plots did not show significant asymmetry, suggesting no potential publication bias for the effect of Armolipid Plus^®^ on the efficacy outcomes (Figures S4–S7). This finding was confirmed by the results of Begg’s rank correlation test and Egger’s linear regression (Table 4).

The Duval and Tweedie trim-and-fill method identified three potentially missing studies on the left side of the funnel plot that resulted in the pooled effect size for DBP reaching statistical significance (Table 4).

### 3.4. Safety Analysis

**Figure 6:**
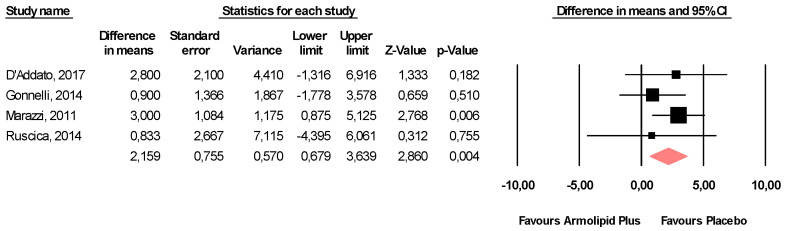
Forest plot displaying mean differences and 95% confidence intervals for the impact of the supplementation with Armolipid Plus^®^ on serum levels of ALT.

Supplementation with Armolipid Plus^®^ exerted a slight, though clinically insignificant, increase in serum levels of ALT (MD = 2.16 U/L, 95%CI(0.68,3.64) U/L, *p* = 0.004; I^2^ = 0%) (Figure 6), without affecting AST (MD = 0.63 U/L, 95%CI(−0.96,2.21) U/L, *p* = 0.437; I^2^ = 0%) or CPK (MD = 7.37 U/L, 95%CI(−1.20,15.93) U/L, *p* = 0.092; I^2^ = 39%) (Figure S8).

Moreover, supplementation with Armolipid Plus^®^ was not associated with increased risk of either musculoskeletal disorders (OR = 0.78, 95%CI(0.29,2.11), *p* = 0.618; I^2^ = 0%) or gastrointestinal disorders (OR = 1.19, 95%CI(0.35,4.06), P = 0.786; I^2^ = 0%) (Figure S9).

The effect sizes were robust in the leave-one-out sensitivity analysis and not mainly driven by a single study (data not shown).

A visual inspection of Begg’s funnel plots did not show significant asymmetry, suggesting no potential publication bias for the effect of Armolipid Plus^®^ on the safety outcomes (Figures S9–S13). This finding was confirmed by the results of Begg’s rank correlation test and Egger’s linear regression (Table 5).

The Duval and Tweedie trim-and-fill method yielded one potentially missing study on the right side of the funnel plot, increasing the pooled effect size for ALT, and one potentially missing study on the right side of the funnel plot, increasing the pooled effect size for CPK. In addition, Duval and Tweedie’s trim-and-fill method yielded one potentially missing study on the left side of the funnel plot, decreasing the pooled effect size for AST, and one potentially missing study on the left side of the funnel plot, decreasing the estimated risk of gastrointestinal disorders (Table 5).

## 4. Discussion

According to our findings, dietary supplementation with Armolipid Plus^®^ exerts a significant effect on BMI and serum levels of TC, TG, HDL-C, LDL-C, hs-CRP, and FPG. Importantly, it is not associated with an increased risk of musculoskeletal symptoms and gastrointestinal disorders, though it results in a slight, though clinically insignificant, increase in ALT serum levels.

To our knowledge, this is the first systematic review and meta-analysis to comprehensively and critically evaluate the existing body of evidence for the use of Armolipid Plus^®^ in daily clinical practice. As a matter of fact, previous meta-analyses on this topic are outdated and not PRISMA compliant [38,39]. Moreover, they included clinical trials that were not adequately controlled for Armolipid Plus^®^ supplementation and clinical studies with an observational design that finally led to not fully reliable results [40,41,42,43,44].

Armolipid Plus^®^ is a dietary supplement widely used in clinical practice and the only combined lipid-lowering nutraceutical recommended by the International Lipid Expert Panel (ILEP) for the management of hypercholesterolemia in statin-intolerant patients [45]. In effect, red yeast rice at the dosage contained in Armolipid Plus^®^ has been shown to be safe also following a recent large meta-analysis of 53 randomized controlled clinical trials enrolling 8535 participants overall [46]. Dietary supplementation with Armolipid Plus^®^ in statin-intolerant patients previously treated with ezetimibe resulted in reductions of approximately 35% in LDL-C and 25% in TG [47], which was similar to results reported for moderate-intensity statins, according to the latest guidelines from the European Society of Cardiology (ESC) and the European Atherosclerosis Society (EAS) [48].

The lipid-lowering effect of red yeast rice (alone or combined with other lipid-lowering nutraceuticals) is well known, as it has been verified by several meta-analyses of randomized controlled clinical trials [49,50]. The interaction between red yeast rice and other natural products with different mechanisms of action, such as the other components of Armolipid Plus^®^, may have additive or synergistic lipid-lowering effects [51]. As a matter of fact, the inhibition of HMG-CoA reductase by monacolins contained in red yeast rice might be advantageously coupled with other nutraceuticals to enhance the hepatic uptake of cholesterol (berberine, soybean proteins), increase lipid excretion in the bowel (soluble fibers, plant sterols, glucomannan, probiotics), or induce LDL-C excretion (berberine, soy proteins, chlorogenic acid) [52,53]. Furthermore, several studies evaluated the efficacy and safety of red yeast rice in combination with policosanols, a mixture of aliphatic alcohols derived from purified sugar cane, even though the mechanism underlying their lipid-lowering effect is still being discussed [54,55]. Policosanols, together with berberine, may also be responsible for the reduction in FPG levels observed after dietary supplementation with Armolipid Plus^®^ [56].

Although there are no trials showing that Armolipid Plus^®^ reduces the risk of ASCVD events, some studies have shown benefits in terms of improved vascular function, as demonstrated by flow-mediated dilation [27] and carotid-femoral pulse wave velocity [57]. Absolute and relative risk reduction (RRR) in CV events with Armolipid Plus^®^ is challenging to estimate based on the available short-term data. There is a linear association between LDL-C reduction and a decrease in ASCVD events, as reported originally by the CTT’s(Cholesterol Treatment Trialists’) meta-analyses of the statin trials where a 1 mmol/L (~39 mg/dL) LDL-C reduction was associated with a 21–23% RRR in CV events over five years [58]. Robust and growing evidence highlights that this linear association is observed regardless of the LDL lowering approach adopted, i.e., low-fat diet, anion exchange resins, ezetimibe, etc. [59]. On the basis of our findings, it is therefore plausible to expect a 14–15% ASCVD event reduction after long-term dietary supplementation with Armolipid Plus^®^.

Despite its strengths, this systematic review and meta-analysis has some limitations. One limitation is the heterogeneity for the effect size on TC and LDL-C, which was moderately high, proving that additional evidence is needed to establish the extent of cholesterol reduction that can be achieved following supplementation with Armolipid Plus^®^. In addition, we had to exclude a relatively large number of clinical trials not compliant with the inclusion criteria for this meta-analysis. The sample size on some laboratory and clinical outcomes was consequently reduced. In particular, some non-significant results (e.g., changes in weight and waist circumference) might be related to a low statistical power.

Nonetheless, the observed results are in line also with a large trial carried out in a setting of the general population involving 1751 volunteers but not included in the meta-analysis, as it did not meet our pre-specified inclusion criteria [42].

Other lipid-lowering nutraceutical combinations could exert a relevant lipid-lowering effect, but the data on Armolipid Plus^®^ are currently most robust.

## 5. Conclusions

Pooling data from the available randomized controlled clinical studies, the current systematic review and meta-analysis provides data in support of the use of Armolipid Plus^®^ in clinical practice as add-on treatment to lifestyle modifications for hypercholesterolemia in order to promote improved cardiometabolic health. Further studies to identify a benefit in terms of CV outcomes are required.

## Figures and Tables

**Table 1:** Main characteristics of the included clinical studies and baseline hemodynamic parameters of enrolled patients.

Author, Year	Study Design	Follow-Up	Main Inclusion Criteria	Study Group	Participants (n)	Background Lipid-Lowering Treatment (Percentage of Subjects)	Male (n (%))	Age (years; mean ± SD)	BMI (Kg/m^2^; mean ± SD)	Waist Circumference (cm; mean ± SD)	SBP (mmHg; mean ± SD)	DBP (mmHg; mean ± SD)
Affuso, 2012 [26]	Randomized, double-blind, placebo-controlled, parallel-group clinical study	18 weeks	Metabolic syndrome 18–65 years of age	Armolipid Plus^®^	29	Statins (28%)	20 (69)	53 ± 7	32.2 ± 4.6	110 ± 9	125 ± 13	78 ± 8
				Placebo	30	Statins (27%)	18 (60)	50 ± 11.9	34.7 ± 5.1	115 ± 13	125 ± 14	81 ± 8
Affuso, 2010 [27]	Randomized, double-blind, placebo-controlled, parallel-group clinical study	6 weeks	18–70 years of age TC > 220 mg/dL LDL-C > 130 mg/dL	Armolipid Plus^®^	25	None	13 (52)	55 ± 8	28 ± 3.8	NA	125 ± 13	78 ± 8
				Placebo	25	None	13 (52)	55 ± 7	28 ± 3.3	NA	125 ± 14	81 ± 8
Cicero, 2012 [28]	Randomized, double-blind, placebo-controlled, parallel-group clinical study	12 months	Primary prevention for CVD overweight	Armolipid Plus^®^	71	None	NA	NA	26.95 ± 0.86	NA	134.4 ± 6.2	86.3 ± 6.1
				Placebo	64	None		NA	24.17 ± 0.99	NA	133.2 ± 5.3	84.1 ± 6.8
D’Addato, 2017 [29]	Multicenter, randomized, double-blind, placebo-controlled, parallel-group clinical study	4 weeks	Primary prevention for CVD 18–75 years of age TC ≥ 200 mg/dL and ≤260 mg/dL LDL-C ≥ 115 mg/dL and ≤180 mg/dL	Armolipid Plus^®^	51	None	17 (33)	53.7 ± 11.6	24 ± 4	NA	NA	NA
				Placebo	51	None	17 (33)	49.7 ± 12.3	24.9 ± 4.6	NA	NA	NA
Galletti, 2019 [30]	Randomized, double-blind, placebo-controlled, parallel-group clinical study	24 weeks	Metabolic syndrome Left ventricular mass >48 g/m^2,7^ for men and >44 g/m^2,7^ for women 18–70 years of age	Armolipid Plus^®^	71	Statins (62%)	42 (59)	55.6 ± 8.9	29.4 ± 3.6	100.8 ± 9.3	130.6 ± 10.5	80.7 ± 8.1
				Placebo	70	Statins (63%)	37 (53)	55.6 ± 9.3	29.2 ± 3.5	100.3 ± 8.7	131.4 ± 10.6	81.6 ± 8
Gentile, 2015 [31]	Randomized, double-blind, placebo-controlled, parallel-group clinical study	8 weeks	Familial combined hyperlipidemia	Armolipid Plus^®^	15	None	(77)	44.1 ± 13	26 ± 2.8	92 ± 10.2	123 ± 12.3	77.9 ± 8.3
				Placebo	15	None			26.7 ± 2.8	97.3 ± 8.5	122.5 ± 9.2	78.1 ± 6.9
Gonnelli, 2014 [32]	Randomized, double-blind, placebo-controlled, parallel-group clinical study	24 weeks	Estimated 10-year CV risk <20% according to Framingham risk scoring 18–60 years of age BMI ≥ 19 Kg/m^2^ and <30 Kg/m^2^ LDL-C > 150 mg/dL	Armolipid Plus^®^	30	None	15 (50)	46.4 ± 9.7	26.9 ± 4.9	89.9 ± 10.9	120.1 ± 11.1	77.2 ± 7
				Placebo	30	None	14 (47)	46.4 ± 10.1	26.4 ± 4.1	88.7 ± 10.9	119.1 ± 19.7	75.2 ± 10
Marazzi, 2017 [33]	Randomized, single-blind, parallel-group clinical study	3 months	Documented CAD treated with PCI in the previous 12 months high-dose statin intolerance LDL-C > 100 mg/dL <50% reduction in LDL-C with low-dose statin treatment	Armolipid Plus^®^ + low-dose statin	50	Statins (100%) Atorvastatin 5 mg (8%) Atorvastatin 10 mg (36%) Simvastatin 10 mg (14%) Simvastatin 20 mg (32%) Rosuvastatin 5 mg (10%)	26 (52)	69 ± 10	NA	NA	NA	NA
				Low-dose statin	50	Statins (100%) Atorvastatin 5 mg (8%) Atorvastatin 10 mg (34%) Simvastatin 10 mg (18%) Simvastatin 20 mg (32%) Rosuvastatin 5 mg (8%)	28 (56)	67 ± 12	NA	NA	NA	NA
Marazzi, 2011 [34]	Randomized, single-blind, placebo-controlled, parallel-group clinical study	12 months	>75 years of age TC> 200 mg/dL LDL-C > 160 mg/dL statin intolerance and refusal of other treatments for hypercholesterolemia	Armolipid Plus^®^	40	None	21 (53)	82.5 ± 4.4	NA	NA	NA	NA
				Placebo	40	None	20 (50)	82.5 ± 4.9	NA	NA	NA	NA
Mercurio, 2020 [35]	Randomized, double-blind, placebo-controlled, parallel-group clinical study	24 weeks	Metabolic syndrome echocardiographic evidence of left ventricular hypertrophy 18–70 years of age stable anti-hypertensive and lipid-lowering therapy over the past three months	Armolipid Plus^®^	79	Statins (55%)	43 (58)	55.6 ± 9	29.1 ± 3	NA	131 ± 11	81 ± 9
				Placebo	79	Statins (58%)	38 (54)	55.6 ± 9	29.3 ± 3	NA	131 ± 11	82 ± 8
Ruscica, 2014 [36]	Randomized, double-blind, placebo-controlled, cross-over clinical study	8 weeks	Primary prevention for CVD metabolic syndrome >18 years of age LDL-C ≥ 130 mg/dL and ≤170 mg/dL	Armolipid Plus^®^	30	None	23 (77)	55.4 ± 9.7	26.8 ± 2.4	96.3 ± 7.9 for men; 91.7 ± 5.1 for women	123 ± 12.3	80.7 ± 5.7
				Placebo								
Solà, 2014 [37]	Randomized, double-blind, placebo-controlled, parallel-group clinical study	12 weeks	Primary prevention for CVD ≥18 years of age LDL-C ≥ 130 mg/dL and <190 mg/dL	Armolipid Plus^®^	51	None	18 (35)	49.9 ± 11.6	25.4 ± 4.1	86.2 ± 11.8	122.2 ± 18.1	76.5 ± 12.2
				Placebo	51	None	14 (28)	52.4 ± 11.2	28 ± 8.7	90.4 ± 11.6	123.8 ± 17.6	76.8 ± 11.2

Expressed as median (interquartile range); BMI = body mass index; CAD = coronary artery disease; CHD = coronary heart disease; CV = cardiovascular; CVD = cardiovascular disease; DBP = diastolic blood pressure; LDL-C = low-density lipoprotein cholesterol; NA = not available; PCI = percutaneous coronary intervention; SBP = systolic blood pressure; SD = standard deviation; TC = total cholesterol.

**Table 2:** Baseline lipids, fasting plasma glucose, and markers of insulin resistance.

Author, Year	Study Group	TC (mg/dL; mean ± SD)	TG (mg/dL; mean ± SD)	HDL-C (mg/dL; mean ± SD)	LDL-C (mg/dL; mean ± SD)	FPG (mg/dL; mean ± SD)	FPI (mU/L; mean ± SD)	HOMA-IR (mean ± SD)	hs-CRP (mg/L; mean ± SD)
Affuso, 2012 [26]	Armolipid Plus^®^	209 ± 39	156 ± 76	42 ± 10	135 ± 7	103 ± 22	9 ± 4.2	3.2 ± 1.5	NA
	Placebo	197 ± 40	170 ± 74	46 ± 14	118 ± 39	85 ± 12	9 ± 6.9	2.7 ± 2.2	NA
Affuso, 2010 [27]	Armolipid Plus^®^	255 ± 29	57 ± 32	58 ± 18	176 ± 25	84 ± 12	NA	NA	NA
	Placebo	252 ± 31	65 ± 28	53 ± 14	171 ± 22	87 ± 12	NA	NA	NA
Cicero, 2012 [28]	Armolipid Plus^®^	218.3 ± 14.4	225.2 ± 42.7	38.6 ± 4.5	134.6 ± 15.2	109.6 ± 12	11.49 ± 4.34	3.2 ± 1.4	2.05 ± 0.31
	Placebo	213.5 ± 17	192.8 ± 44.4	39 ± 4.3	136 ± 18.9	92.2 ± 10.3	7.47 ± 3.14	1.7 ± 0.8	1.85 ± 0.43
D’Addato, 2017 [29]	Armolipid Plus^®^	234.6 ± 18	110.8 ± 41.5	65.1 ± 13.3	147.5 ± 16.3	NA	NA	NA	NA
	Placebo	235.6 ± 17.9	110.5 ± 41.9	70 ± 16.2	143.6 ± 15	NA	NA	NA	NA
Galletti, 2019 [30]	Armolipid Plus^®^	224.3 ± 44.7	151.3 ± 82.5	50.7 ± 11.9	132.9 ± 36.5	103.9 ± 14.5	15.7 ± 11.6	4.1 ± 3.2	1.85 ± 2.34
	Placebo	218.4 ± 38.2	159.6 ± 86.6	50.4 ± 12.1	128.4 ± 28.6	105.7 ± 17.9	16.3 ± 9	4.2 ± 2.4	1.35 ± 1.01
Gentile, 2015 [31]	Armolipid Plus^®^	228.8 ± 41.1	290.3 ± 104.3	40.8 ± 6.6	134.7 ± 46.5	91.5 ± 17.5	NA	NA	NA
	Placebo	241.9 ± 42.1	204.2 ± 80.9	38.2 ± 9.1	162.8 ± 41.2	93 ± 5.9	NA	NA	NA
Gonnelli, 2014 [32]	Armolipid Plus^®^	238.4 ± 26.9	132.1 ± 55.2	53.1 ± 13.2	162 ± 22.5	92.5 ± 8.8	NA	NA	NA
	Placebo	248.1 ± 32.4	119 ± 50.4	55.7 ± 14.5	165.8 ± 29	94.4 ± 10	NA	NA	NA
Marazzi, 2017 [33]	Armolipid Plus^®^	198 ± 9	177 ± 51	35 ± 4	127 ± 15	NA	NA	NA	NA
	Placebo	199 ± 11	176 ± 51	35 ± 4	129 ± 17	NA	NA	NA	NA
Marazzi, 2011 [34]	Armolipid Plus^®^	252 ± 23	179 ± 48	44 ± 12	172 ± 16	94 ± 6	7.2 ± 2.4	1.7 ± 0.6	NA
	Placebo	253 ± 19	179 ± 50	44 ± 8	173 ± 10	91 ± 7	6.5 ± 2.4	1.5 ± 0.6	NA
Mercurio, 2020 [35]	Armolipid Plus^®^	227 ± 44	160 ± 88	49 ± 11	138 ± 34	105 ± 16	NA	4.2 ± 3	NA
	Placebo	218 ± 40	151 ± 83	53 ± 13	124 ± 30	104 ± 16	NA	4 ± 3	NA
Ruscica, 2014 [36]	Armolipid Plus^®^	240 ± 31	216 (171, 284)*	40 ± 9	151 ± 24	88 ± 16	6 ± 4	1.3 ± 0.9	2 ± 1
	Placebo	240 ± 39	230 (173, 307)*	41 ± 7	150 ± 29	86 ± 18	6.4 ± 4.4	1.3 ± 1	2 ± 3
Solà, 2014 [37]	Armolipid Plus^®^	243.6 ± 24.4	107.2 ± 61.3	66.5 ± 21.2	155.7 ± 14.6	90.6 ± 9.3	8.2 ± 9.2	1.8 ± 2.6	NA
	Placebo	243.4 ± 19.5	115 ± 56	61.1 ± 14.1	159.3 ± 15.7	92.8 ± 10.3	7.5 ± 5.4	1.7 ± 1.3	NA

Expressed as median (interquartile range); FPG = fasting plasma glucose; FPI =fasting plasma insulin; HDL-C = high-density lipoprotein cholesterol; HOMA-IR = homeostatic model assessment for insulin resistance; hs-CRP = high sensitivity C reactive protein; LDL-C = low-density lipoprotein cholesterol; NA = not available; SD = standard deviation; TC = total cholesterol; TG = triglycerides.

**Table 3:** Quality of bias assessment of the included studies according to the Cochrane guidelines.

First Author, Year	Sequence Generation	Allocation Concealment	Blinding of Participants, Personnel, and Outcome Assessment	Incomplete Outcome Data	Selective Outcome Reporting	Other Potential Threats to Validity
Affuso, 2012 [26]	L	L	L	L	L	U
Affuso, 2010 [27]	L	L	L	L	L	U
Cicero, 2012 [28]	L	L	L	L	L	L
D’Addato, 2017 [29]	L	L	L	L	L	L
Galletti, 2019 [30]	L	L	L	L	L	L
Gentile, 2015 [31]	L	L	L	L	L	U
Gonnelli, 2014 [32]	L	L	L	L	L	U
Marazzi, 2017 [33]	L	L	L	L	L	L
Marazzi, 2011 [34]	L	L	L	L	L	U
Mercurio, 2020 [35]	L	L	L	L	L	L
Ruscica, 2014 [36]	L	L	L	L	L	U
Solà, 2014 [37]	L	L	L	L	L	L

H = High risk of bias; L = Low risk of bias; U = Unclear risk of bias.

**Table 4:** Assessment of publication bias on efficacy outcomes.

Outcomes	Adjustment with Duval and Tweedie’s Trim-and-Fill Method				Begg’s Rank Correlation Test	Egger’s Linear Regression
	Number of Trimmed Studies	Adjusted Effect Sizes				
		MD	95% Confidence Interval		*p*-Value	*p*-Value
			Lower Bound	Upper Bound		
Weight	-	-	-	-	0.602	0.672
BMI	2	−0.262	−0.440	−0.085	1	0.174
Waist circumference	1	−0.819	−3.10	1.462	0.174	0.6
SBP	2	1.044	−1.287	3.374	0.624	0.485
DBP	3	−1.764	−3.192	−0.336	0.624	0.186
TC	1	−26.708	−29.212	−24.203	0.815	0.981
TG	2	−10.559	−16.861	−4.257	0.337	0.238
HDL-C	2	1.658	0.778	2.537	0.484	0.587
LDL-C	2	−29.049	−31.667	−26.432	0.392	0.478
FPG	2	−4.007	−5.521	−2.492	0.532	0.563
FPI	-	-	-	-	0.652	0.842
HOMA-IR	-	-	-	-	0.851	0.852

BMI = body mass index; DBP = diastolic blood pressure; FPG = fasting plasma glucose; FPI = fasting plasma insulin; HDL-C = high-density lipoprotein cholesterol; HOMA-IR = homeostatic model assessment for insulin resistance; LDL-C = low-density lipoprotein cholesterol; MD = mean difference; SBP = systolic blood pressure; TC = total cholesterol; TG = triglycerides.

**Table 5:** Assessment of publication bias on safety outcomes.

Safety Parameters	Adjustment with Duval and Tweedie’s Trim-and-Fill Method				Begg’s Rank Correlation Test	Egger’s Linear Regression
	Number of Trimmed Studies	Adjusted Effect Sizes				
		MD	95% Confidence Interval		*p*-Value	*p*-Value
			Lower Bound	Upper Bound		
ALT	1	2.275	0.851	3.699	0.497	0.611
AST	1	0.583	−0.983	2.15	0.497	0.601
CPK	1	7.868	− 0.365	16.101	0.174	0.552
**Treatment-Emergent Adverse Events**	**Adjustment with Duval and Tweedie’s Trim-and-Fill Method**				**Begg’s Rank Correlation Test**	**Egger’s Linear Regression**
	**Number of Trimmed Studies**	**Adjusted Effect Sizes**				
		**OR**	**95% Confidence Interval**		***p*-Value**	***p*-Value**
			**Lower Bound**	**Upper Bound**		
Musculoskeletal disorders	-	-	-	-	1	0.759
Gastrointestinal disorders	1	1.014	0.321	3.201	0.602	0.951

ALT = alanine aminotransferase; AST = aspartate aminotransferase; CPK = creatine phosphokinase; MD = mean difference; OR = odds ratio.

## Data Availability

Data supporting findings of this analysis are available from the Corresponding Authors upon reasonable request.

## Supplementary Materials

The following are available online at https://www.mdpi.com/2072-6643/13/2/638/s1, Figure S1: Forest plot displaying mean differences and 95% confidence intervals for the impact of supplementation with Armolipid Plus^®^ on weight and WC, Figure S2: Forest plot displaying mean differences and 95% confidence intervals for the impact of supplementation with Armolipid Plus^®^ on SBP and DBP, Figure S3: Forest plot displaying mean differences and 95% confidence intervals for the impact of supplementation with Armolipid Plus^®^ on FPI and HOMA-IR, Figure S4: Funnel plots detailing publication bias for the effect of supplementation with Armolipid Plus^®^ on weight, BMI, and waist circumference, Figure S5: Funnel plots detailing publication bias for the effect of supplementation with Armolipid Plus^®^ on blood pressure, Figure S6: Funnel plots detailing publication bias for the effect of supplementation with Armolipid Plus^®^ on serum lipid concentrations, Figure S7: Funnel plots detailing publication bias for the effect of supplementation with Armolipid Plus^®^ on glycemia and markers of insulin resistance, Figure S8: Forest plot displaying mean differences and 95% confidence intervals for the impact of supplementation with Armolipid Plus^®^ on AST and CPK, Figure S9: Forest plots displaying the risk of treatment-emergent adverse events during supplementation with Armolipid Plus^®^, Figure S10: Funnel plot detailing publication bias for the effect of supplementation with Armolipid Plus^®^ on serum concentrations of ALT, Figure S11: Funnel plot detailing publication bias for the effect of supplementation with Armolipid Plus^®^ on serum concentrations of AST, Figure S12: Funnel plot detailing publication bias for the effect of supplementation with Armolipid Plus^®^ on serum concentrations of CPK, Figure S13: Funnel plot detailing publication bias for risk of treatment-emergent adverse events during supplementation with Armolipid Plus^®^.
