# Supplementary material for: Efficacy and Safety of Armolipid Plus®: An Updated PRISMA Compliant Systematic Review and Meta-Analysis of Randomized Controlled Clinical Trials
Source: Nutrients. 2021 Feb 16;13(2):638. doi: 10.3390/nu13020638 (PMC7920267; doi:10.3390/nu13020638)

SUPPLEMENTARY MATERIALS

Figure S1. Forest plot displaying mean differences and 95% confidence intervals for the impact of supplementation with Armolipid Plus® on weight and WC.

Weight

Study name	Difference in means	Standard error	Variance	Lower limit	Upper limit	Z-Value	p-Value
Affuso, 2012	-1,000	4,051	16,409	-8,939	6,939	-0,247	0,805
Gonnelli, 2014	0,700	3,392	11,504	-5,948	7,348	0,206	0,836
Solà, 2014	-1,890	2,759	7,614	-7,298	3,518	-0,685	0,493
	-0,889	1,893	3,582	-4,599	2,820	-0,470	0,638

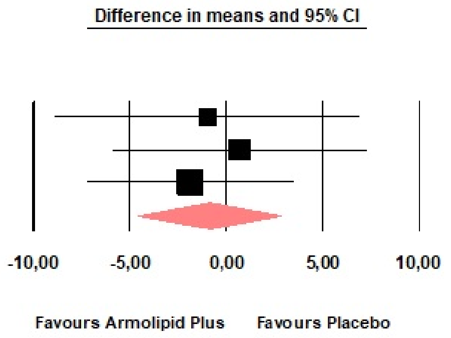

Waist Circumference

Study name	Difference in means	Standard error	Variance	Lower limit	Upper limit	Z-Value	p-Value
Affuso, 2012	-2,000	2,921	8,529	-7,724	3,724	-0,685	0,493
Gentile, 2015	-0,200	3,416	11,667	-6,895	6,495	-0,059	0,953
Gonnelli, 2014	-0,200	2,691	7,244	-5,475	5,075	-0,074	0,941
Solà, 2014	0,050	2,244	5,033	-4,347	4,447	0,022	0,982
	-0,499	1,361	1,853	-3,167	2,169	-0,367	0,714

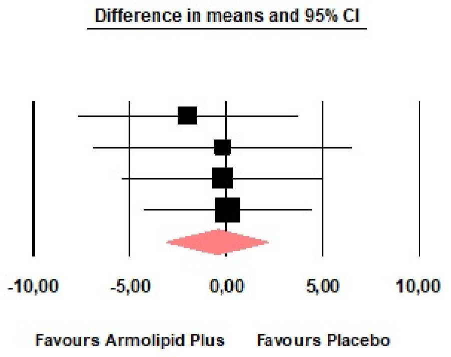

Figure S2. Forest plot displaying mean differences and 95% confidence intervals for the impact of supplementation with Armolipid Plus® on SBP and DBP.

Systolic Blood Pressure

Study name	Difference in means	Standard error	Variance	Lower limit	Upper limit	Z-Value	p-Value
Affuso, 2012	-6,000	3,219	10,365	-12,310	0,310	-1,864	0,062
Gentile, 2015	-0,900	4,374	19,131	-9,473	7,673	-0,206	0,837
Gonnelli, 2014	-2,900	4,256	18,111	-11,241	5,441	-0,681	0,496
Mercurio, 2020	1,000	1,925	3,706	-2,773	4,773	0,519	0,603
Solà, 2014	2,430	3,496	12,219	-4,421	9,281	0,695	0,487
	-0,571	1,342	1,800	-3,200	2,058	-0,426	0,670

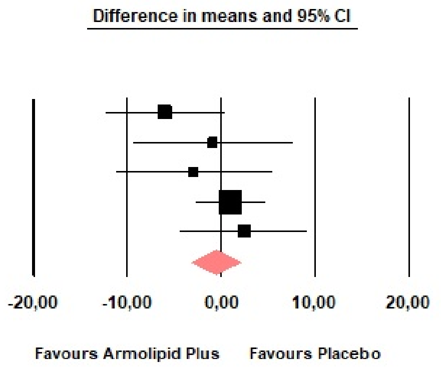

Diastolic Blood Pressure

Study name	Difference in means	Standard error	Variance	Lower limit	Upper limit	Z-Value	p-Value
Affuso, 2012	0,000	2,025	4,099	-3,968	3,968	0,000	1,000
Gentile, 2015	-0,500	2,763	7,635	-5,916	4,916	-0,181	0,856
Gonnelli, 2014	-1,100	2,308	5,326	-5,623	3,423	-0,477	0,634
Mercurio, 2020	-2,000	1,376	1,893	-4,697	0,697	-1,454	0,146
Solà, 2014	0,840	2,189	4,791	-3,450	5,130	0,384	0,701
	-0,887	0,877	0,769	-2,607	0,832	-1,012	0,312

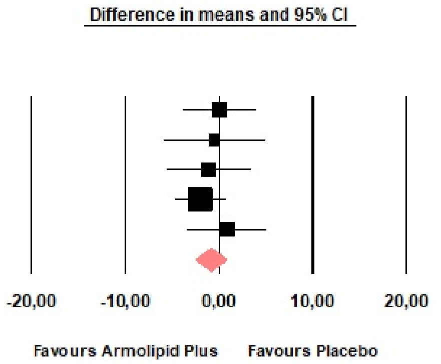

Figure S3. Forest plot displaying mean differences and 95% confidence intervals for the impact of supplementation with Armolipid Plus® on FPI and HOMA-IR.

Fasting Plasma Insulin

Study name	Difference in means	Standard error	Variance	Lower limit	Upper limit	Z-Value	p-Value
Affuso, 2012	-3,500	1,237	1,529	-5,924	-1,076	-2,830	0,005
Affuso, 2010	0,100	1,371	1,879	-2,586	2,786	0,073	0,942
Cicero, 2012	-0,800	0,780	0,609	-2,329	0,729	-1,026	0,305
Marazzi, 2011	-0,400	0,503	0,253	-1,386	0,586	-0,795	0,427
Mercurio, 2020	1,400	1,839	3,382	-2,204	5,004	0,761	0,446
Ruscica, 2014	0,400	0,971	0,943	-1,504	2,304	0,412	0,680
Solà, 2014	-1,400	1,447	2,093	-4,236	1,436	-0,968	0,333
	-0,576	0,341	0,116	-1,244	0,092	-1,690	0,091

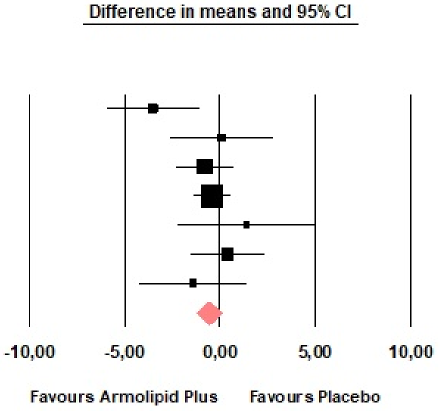

HOMA-IR

Study name	Difference in means	Standard error	Variance	Lower limit	Upper limit	Z-Value	p-Value
Affuso, 2012	-1,100	0,415	0,173	-1,914	-0,286	-2,648	0,008
Cicero, 2012	0,400	0,241	0,058	-0,072	0,872	1,660	0,097
Marazzi, 2011	-0,170	0,130	0,017	-0,424	0,084	-1,313	0,189
Mercurio, 2020	0,360	0,503	0,253	-0,625	1,345	0,716	0,474
Ruscica, 2014	0,100	0,222	0,049	-0,335	0,535	0,450	0,653
Solà, 2014	-0,430	0,388	0,151	-1,191	0,331	-1,108	0,268
	-0,094	0,178	0,032	-0,443	0,255	-0,526	0,599

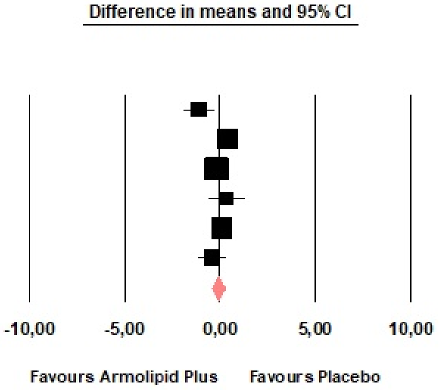

Figure S4. Funnel plots detailing publication bias for the effect of supplementation with Armolipid Plus® on weight, BMI and waist circumference.

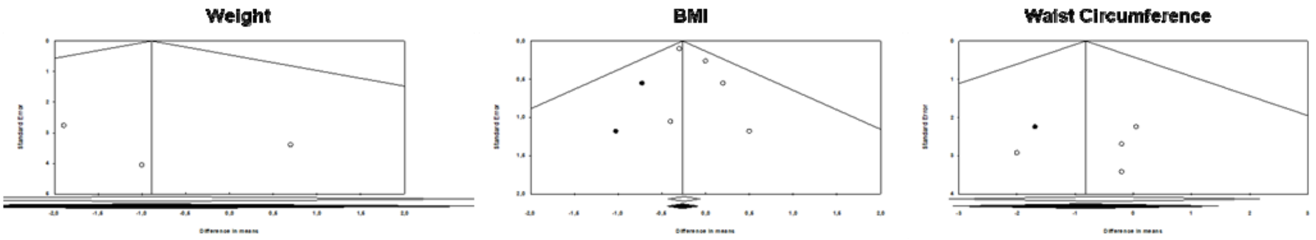

Figure S5. Funnel plots detailing publication bias for the effect of supplementation with Armolipid Plus® on blood pressure.

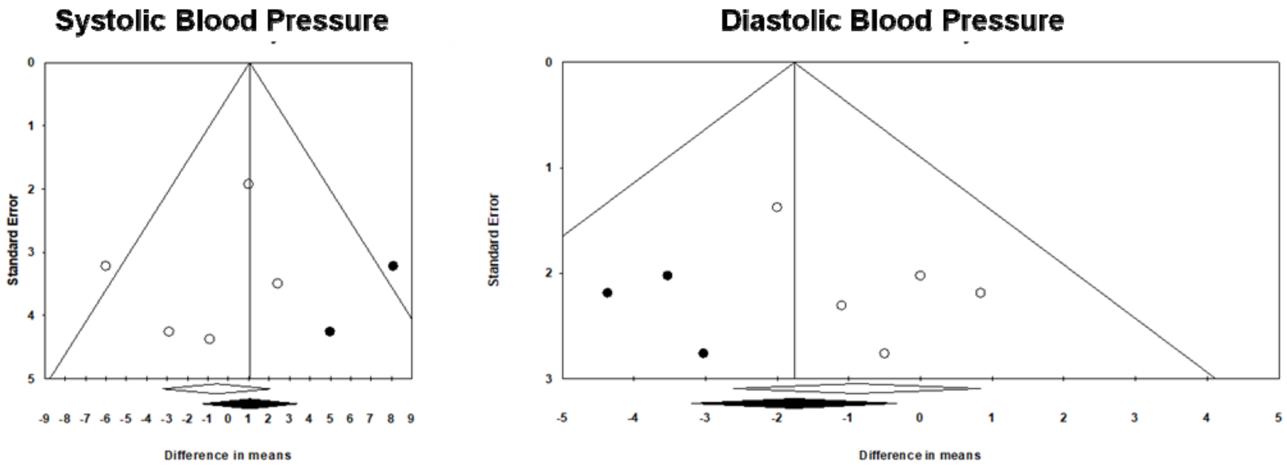

Figure S6. Funnel plots detailing publication bias for the effect of supplementation with Armolipid Plus® on serum lipids concentrations.

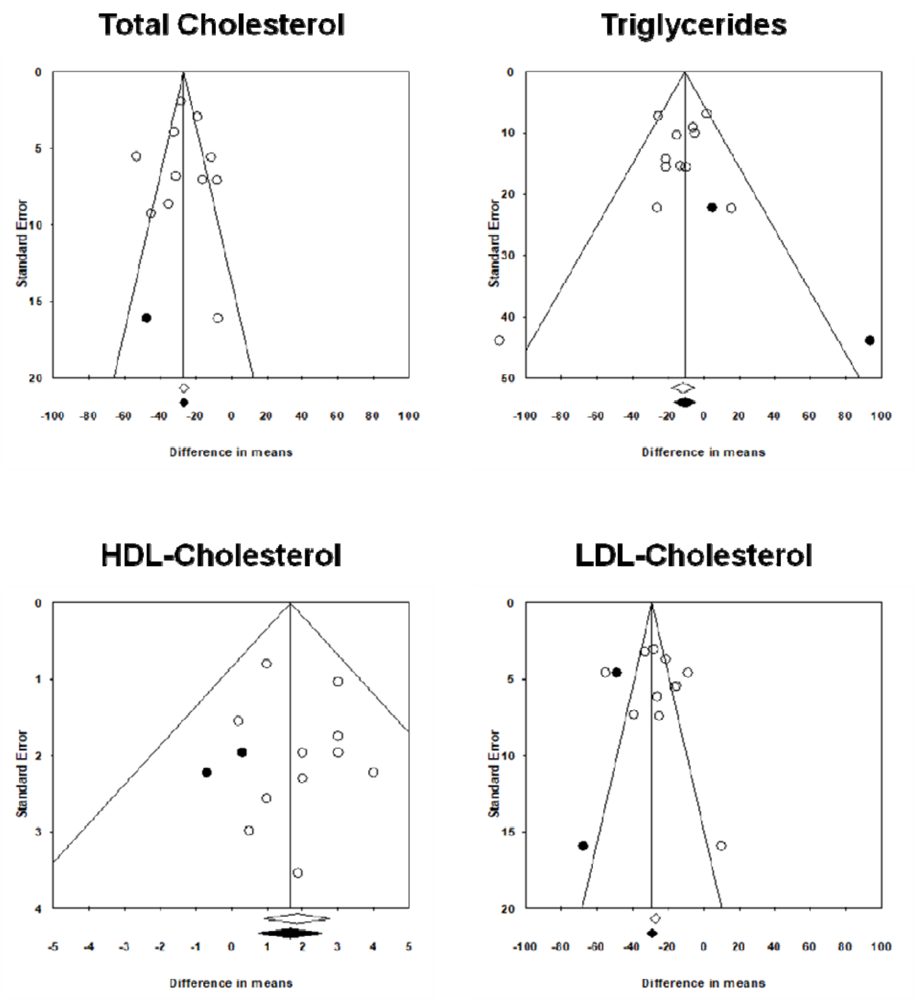

Figure S7. Funnel plots detailing publication bias for the effect of supplementation with Armolipid Plus® on glycemia and markers of insulin resistance.

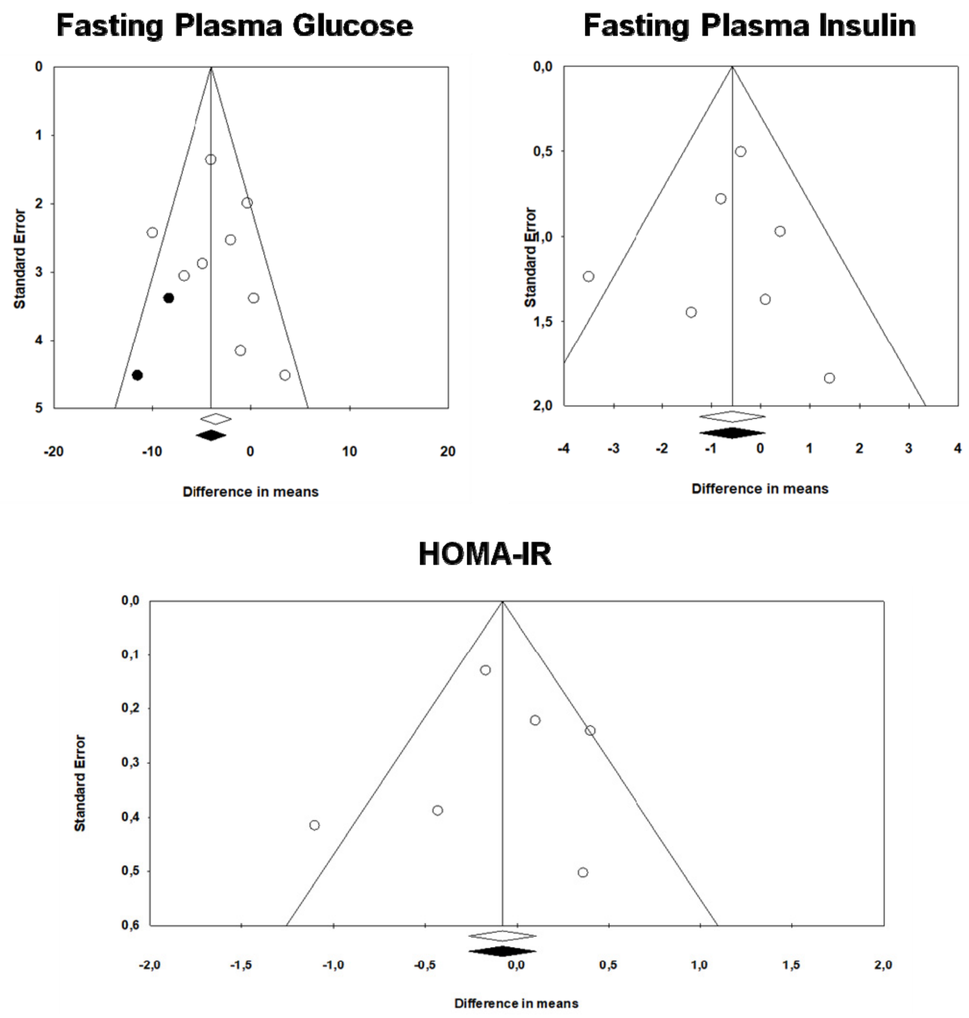

Figure S8. Forest plot displaying mean differences and 95% confidence intervals for the impact of supplementation with Armolipid Plus® on AST and CPK.

Aspartate Aminotransferase

Study name	Difference in means	Standard error	Variance	Lower limit	Upper limit	Z-Value	p-Value
D'Addato, 2017	0,900	1,594	2,540	-2,224	4,024	0,565	0,572
Gonnelli, 2014	-3,700	2,840	8,063	-9,265	1,865	-1,303	0,193
Marazzi, 2011	1,000	1,012	1,025	-0,984	2,984	0,988	0,323
Ruscica, 2014	2,500	5,267	27,746	-7,824	12,824	0,475	0,635
	0,628	0,809	0,654	-0,956	2,213	0,777	0,437

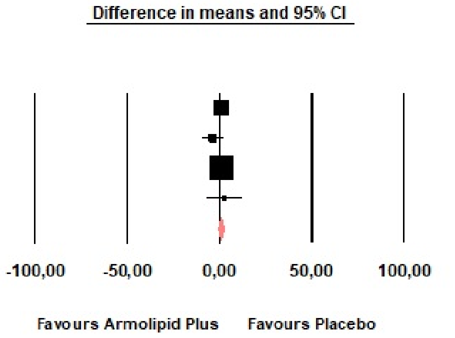

Creatine Phosphokinase

Study name	Difference in means	Standard error	Variance	Lower limit	Upper limit	Z-Value	p-Value
D'Addato, 2017	3,200	14,349	205,893	-24,923	31,323	0,223	0,824
Gonnelli, 2014	39,900	15,421	237,797	9,676	70,124	2,587	0,010
Marazzi, 2011	5,000	5,060	25,600	-4,917	14,917	0,988	0,323
Ruscica, 2014	1,700	15,292	233,848	-28,272	31,672	0,111	0,911
	7,365	4,368	19,084	-1,197	15,927	1,686	0,092

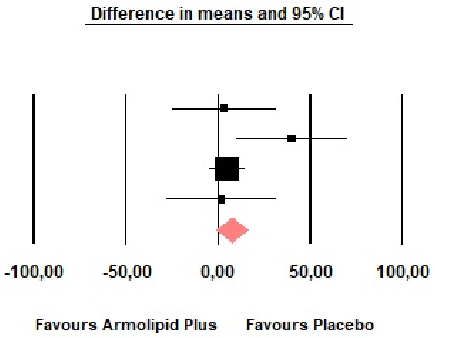

Figure S9. Forest plots displaying the risk of treatment-emergent adverse events during supplementation with Armolipid Plus®.

Musculoskeletal Disorders

Study name	Statistics for each study					Events / Total	
	Odds ratio	Lower limit	Upper limit	Z-Value	p-Value	Armolipid Plus	Placebo
Gonnelli, 2014	0,322	0,013	8,235	-0,685	0,494	0 / 30	1 / 30
Marazzi, 2017	1,000	0,192	5,210	0,000	1,000	3 / 50	3 / 50
Marazzi, 2011	0,568	0,126	2,554	-0,738	0,460	3 / 40	5 / 40
Mercurio, 2020	2,918	0,117	72,831	0,652	0,514	1 / 74	0 / 71
	0,775	0,285	2,107	-0,499	0,618		

Odds ratio and 95% CI

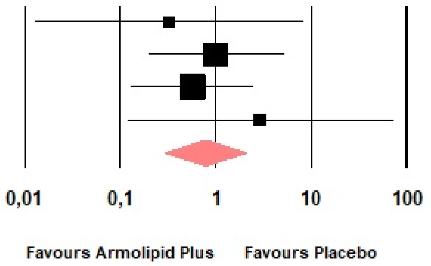

Gastrointestinal Disorders

Study name	Statistics for each study					Events / Total	
	Odds ratio	Lower limit	Upper limit	Z-Value	p-Value	Armolipid Plus	Placebo
Galletti, 2019	2,958	0,118	73,847	0,661	0,509	1 / 72	0 / 70
Gonnelli, 2014	1,385	0,282	6,796	0,401	0,688	4 / 30	3 / 30
Marazzi, 2017	0,490	0,043	5,582	-0,575	0,565	1 / 50	2 / 50
	1,186	0,347	4,060	0,272	0,786		

Odds ratio and 95% CI

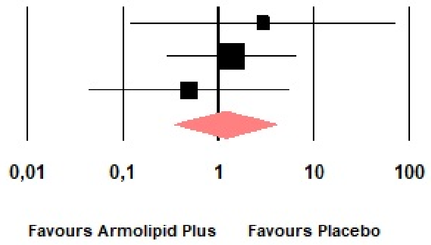

Figure S10. Funnel plot detailing publication bias for the effect of supplementation with Armolipid Plus® on serum concentrations of ALT.

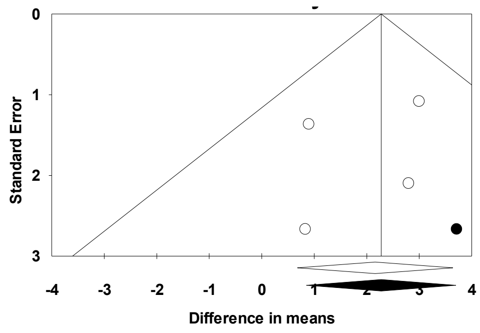

Figure S11. Funnel plot detailing publication bias for the effect of supplementation with Armolipid Plus® on serum concentrations of AST.

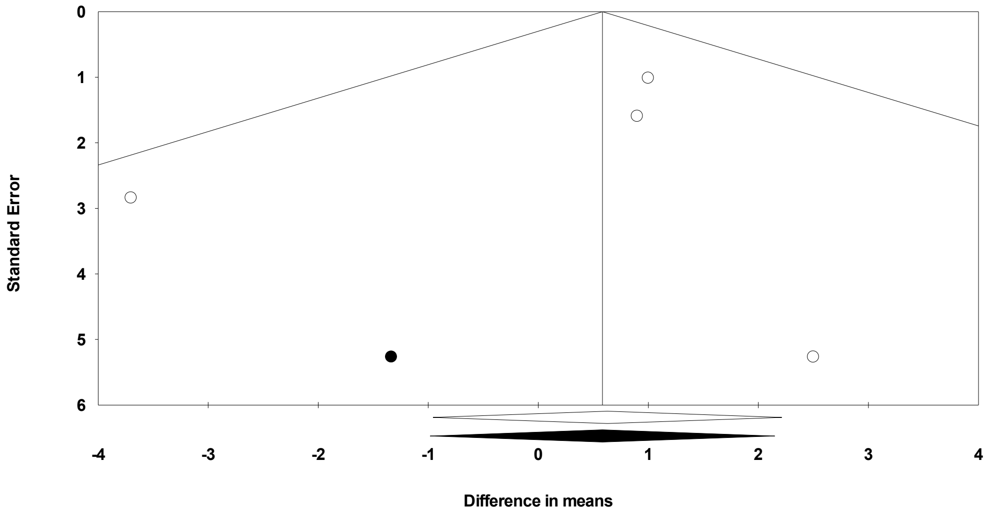

Figure S12. Funnel plot detailing publication bias for the effect of supplementation with Armolipid Plus® on serum concentrations of CPK.

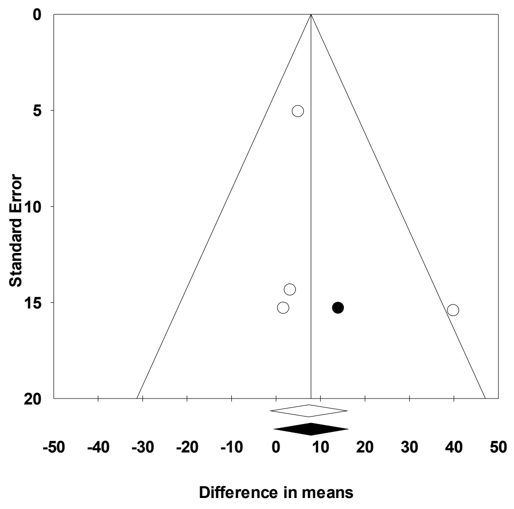

Figure S13. Funnel plot detailing publication bias for risk of treatment-emergent adverse events during supplementation with Armolipid Plus®.

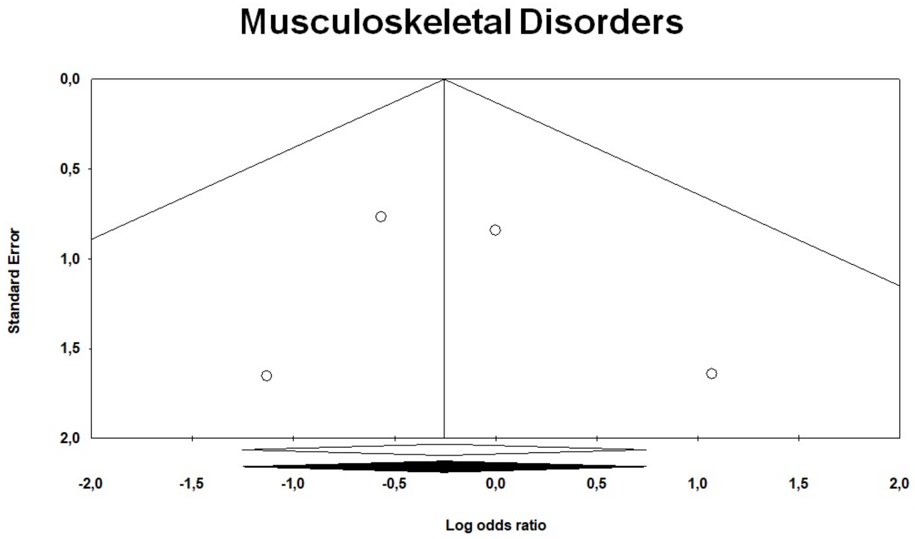

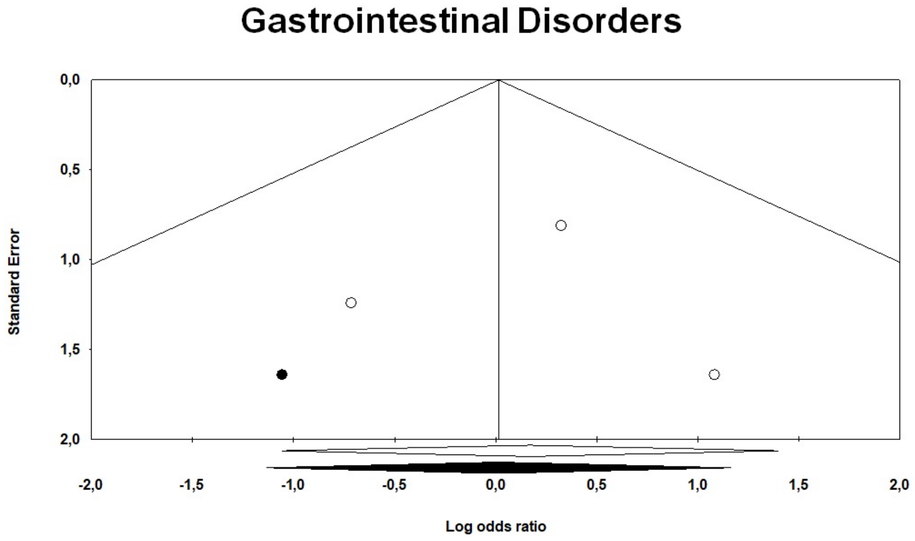

Supplement: Supplementary file 1 [file nutrients-13-00638-s001.pdf]
